# Optimization of Dissolution Parameters for GH4738 Scrap via Response Surface Methodology

**DOI:** 10.3390/ma18040793

**Published:** 2025-02-11

**Authors:** Guiqun Liu, Xinyu Fang, Xiaoli Zhang, Guanglei Lv

**Affiliations:** 1 School of Material Science and Engineering, North Minzu University, Yinchuan 750021, China; gqliu10b@alum.imr.ac.cn (G.L.); fxy166886886@163.com (X.F.); 2 CNOOC (Tianjin) Pipeline Engineering Technology Ltd., Tianjin 300452, China; lvgl2@cnooc.com.cn

**Keywords:** Ni-based superalloy, dissolution rate, energy consumption, Plackett–Burman design, response surface methodology

## Abstract

This study aimed to optimize the electrochemical dissolution process of GH4738 scrap, a Ni-based superalloy, to achieve a high dissolution rate with minimal energy consumption. Using the Plackett–Burman design, we identified four key factors from a pool of eight candidates that significantly influence both the dissolution rate and energy consumption: current density, NiCl_2_ concentration, electrolysis time, and H_2_SO_4_ concentration. The steepest ascent method was then applied to define a region that minimized energy consumption while maximizing the dissolution rate. Response surface methodology (RSM) was used to determine the central point for further analysis, providing valuable insights for optimizing the dissolution parameters. The study demonstrated that increasing the NiCl_2_ concentration reduced the breakdown potential, and at an H_2_SO_4_ concentration of 1.5 mol/L, high dissolution efficiency was achieved with minimal energy consumption. The interactions among the parameters significantly affected the dissolution performance. Analysis of variance (ANOVA) confirmed the significant influence of these parameters on the dissolution behavior of Ni-based superalloys. This research contributes to the understanding of GH4738 scrap dissolution and provides a systematic approach for optimizing the process, which is crucial for efficient material recovery and laboratory sustainability.

## 1. Introduction

Industrialization has rendered non-ferrous metals essential across economic, societal, defense, and technological sectors [1]. Their increasing demand underscores the critical role they play in societal progress. Recycling scrap metals and promoting their circular use are strategic moves aimed at conserving resources, reducing reliance on external sources, and ensuring economic sustainability. Improving recycling efficiency is crucial for maintaining a sustainable industry, safeguarding national security, and preserving ecological balance [2,3]. In the field of metal recycling, pyrometallurgy and hydrometallurgy are key methods for extracting metals from secondary sources. A comprehensive review of the literature reveals that pyrometallurgy, which employs high-temperature processes to separate metals from impurities, is known for its simplicity and high recovery rates [4]. However, hydrometallurgy, which includes leaching and solvent extraction, is often considered a more sustainable approach compared to traditional methods. This is due to its reduced environmental impact and potentially higher recovery rates [5,6,7]. For instance, Guan [5] reported a 79.40% leaching rate using hydrochloric acid on coal gangue, while Yang [6] achieved a 94.68% extraction rate with an aqueous process. Ding [7] showed that ultrasonic assistance can markedly improve leaching efficiency, raising zinc leaching rates from 94.43% to 99.57%.

In practical production and applications, the dissolution behavior of GH4738 scrap is subject to increasingly complex laboratory conditions. However, most current research has focused on single-variable conditions, which limits our understanding of the combined effects of these variables on the dissolution of Ni-based superalloys [8]. Furthermore, there is a lack of systematic statistical analysis on the dissolution response of GH4738 scrap. To address this gap, this study applied Response Surface Methodology (RSM) to statistically analyze the dissolution behavior of GH4738 scrap. The study investigated the impact of NiCl_2_ concentration, current density, H_2_SO_4_ concentration, and electrolysis time on both the dissolution rate (η) and energy consumption (E) of GH4738 scrap using Design-Expert 13.0.5.0. The RSM experimental design facilitated the analysis of parameter contributions and their interactions, effectively fitting a quadratic surface model to optimize the dissolution parameters. This study aims to optimize the dissolution parameters of GH4738 scrap using Response Surface Methodology to enhance dissolution efficiency and reduce energy consumption. By analyzing multiple variables, this research provides a scientific basis for the efficient recycling and reuse of GH4738 scrap.

## 2. Experimental

### 2.1. Composition of GH4738 Scrap Material

GH4738 is a nickel-based superalloy renowned for its exceptional mechanical properties at high temperatures. This makes it widely used in the aerospace and power-generation sectors. However, the production and fabrication of GH4738 components generate a significant amount of scrap material. The scrap used in this study was sourced from KOCEL Steel Foundry Co., Ltd. (Yinchuan, China), a leading Chinese company specializing in large-scale steel castings. The company is well known for manufacturing high-quality castings for energy equipment, including gas turbines, steam turbines, and hydroelectric power components. The chemical composition of these scraps matches that of the original GH4738 alloy, as shown in Table 1.

### 2.2. Performance Testing Method

An electrochemical workstation was used to measure the anodic polarization curve, electrochemical impedance spectroscopy (EIS), and Tafel curve of electrolytic nickel in the electrolyte. The detailed procedure is as follows:

(a) Sample Preparation: The sample was polished with abrasive papers of sequentially finer grit sizes: 400#, 600#, 1000#, 1500#, and 2000#. This was performed to ensure a consistent surface finish for each sample. (The samples utilized in this study were derived from GH4738 scrap material. To ensure uniformity and ease of handling, the samples were machined into a parallelepiped form, with each face having a surface area of 1 cm^2^. This geometric configuration was selected to facilitate consistent processing and subsequent analysis. Following machining, the samples were embedded in epoxy resin to protect the edges and provide a stable base for further testing and characterization.)
(b) Electrochemical Setup: In the three-electrode setup, the sample acted as the working electrode, with a titanium electrode functioning as the auxiliary electrode in a loop configuration. A saturated calomel electrode served as the reference electrode, providing the potential for the working electrode and establishing the potential standard for the system [9].
(c) Electrolysis and Measurement: Post-electrolysis, the anode residual material was dried and weighed to calculate the dissolution rate using the formula outlined below [10]:

(1) $$\eta =\frac{g_{o}-g_{1}}{g_{o}}\times 100\%$$

where *η* is alloy dissolution rate [%], *g_0_* is the nickel anode weigh [g], and *g_1_* is the nickel anode residual weight [g].

(d) Energy Consumption Calculation: The calculation formula for the energy consumption of GH4738 scrap dissolution is as follows [11]:

(2) $$E=\frac{UIt}{∆m}$$

where *E* is the electrolysis energy consumption [kW·h·g^−1^], *U* is the tank voltage [V], *I* is the current [A], and *∆m* is the experimental weight loss [g].

### 2.3. Response Surface Methodology

#### 2.3.1. Plackett–Burman Design

The Plackett–Burman Design (PBD) incorporated eight critical factors, which were identified following a review of single-factor experiments. Spanning 12 experimental groups, the design featured eight active factors and three placeholders, with four groups designated as blank controls. Factors were assessed at two levels—high and low—to determine their impact. The study’s objectives were to quantify dissolution rate and energy consumption. Table 2 outlines the factors and their levels within the PBD.

The Plackett–Burman (PB) experiment delineated the path of steepest ascent, aligning with the gradient to efficiently reach the optimal value range. The experiment identified current density, H_2_SO_4_ concentration, NiCl_2_ concentration (the use of NiCl_2_ as an additive is favored for its ability to provide nickel cations, which positively influence the electrochemical dissolution process. Moreover, NiCl_2_ is more effective than HCl in reducing energy consumption and enhancing dissolution efficiency.), and electrolysis time as pivotal factors. The subsequent steepest ascent test targeted these factors.

#### 2.3.2. Path of the Steepest Ascent Method

The results from the Plackett–Burman (PB) experiment enabled the identification of the direction of the steepest ascent, which is crucial for efficiently targeting the optimal value region. The experiment pinpointed current density, H_2_SO_4_ concentration, NiCl_2_ concentration, and electrolysis time as the key factors affecting the process. Subsequently, the steepest ascent test was performed focusing on these factors.

#### 2.3.3. Box–Behnken Design

Response Surface Methodology (RSM) is utilized to validate and refine the empirical model’s design parameters, employing polynomial equations derived from experimental data. This approach effectively identifies the factors that influence outcomes and clarifies the process of data analysis. The Central Composite Design (CCD)-based RSM model, as implemented in Design Expert version 13 software, was applied to evaluate the parameters for dissolution testing. RSM excels in analyzing parameter interactions and optimizing system parameters efficiently with a minimal number of tests. As outlined in Table 3, the study focused on four parameters: current density, H_2_SO_4_ concentration, NiCl_2_ concentration, and electrolysis time, each tested across three levels. A total of twenty-nine experimental runs were conducted to measure the responses of these parameters. The energy consumption and dissolution rate data were modeled using a standard second-order polynomial equation, as referenced in [11].

(3) $$R=a_{0}+a_{1}x_{1}+a_{2}x_{2}+a_{3}x_{3}+a_{4}x_{4}+a_{5}x_{1}^{2}+a_{6}x_{2}^{2}+a_{7}x_{3}^{2}+a_{8}x_{4}^{2}+a_{9}x_{1}x_{2}+a_{10}x_{1}x_{3}+a_{11}x_{1}x_{4}+a_{12}x_{2}x_{3}+a_{13}x_{2}x_{4}+a_{14}x_{3}x_{4}$$

## 3. Results

### 3.1. Corrosion Reaction

Drawing from the established knowledge of nickel (Ni) and its superalloys’ corrosion mechanisms as detailed in prior research [12,13], the general reaction for Ni corrosion in chloride-containing solutions is depicted by the subsequent equations:

(4) $$Ni{\left(HO\right)}_{2}+NiO+{6Cl}^{-}\to 3Ni{Cl}_{2}+O_{2}+2H_{2}O+{2OH}^{-}+4e^{-}$$

The anode dissolution:

(5) $$NiOH^{+}+2Cl^{-}\to NiCl_{2}+{OH}^{-}$$

The anode passivation:

(6) $$2NiO+4{Cl}^{-}\to 2Ni{Cl}_{2}+O_{2}+{4e}^{-}$$

### 3.2. Result of Plackett–Burman Design

This study utilized the Plackett–Burman design (PBD) with 12 trials to assess the impact of eight factors, derived from single-factor testing, on the dissolution rate and energy consumption. Each factor was evaluated at two levels: high and low. To accommodate experimental variability, four dummy variables were included. Table 4 details the factors and their respective levels. Design Expert 13 software facilitated the analysis of results, comparing the influence of each factor on dissolution rate (η) and energy consumption (E), and identified the most significant factors influencing the process.

The Plackett–Burman (PB) test’s main effect analysis for each influencing factor is detailed in Table 5. The model’s *p*-value (the *p*-value is used to assess the significance of each factor’s effect on the response variables) of 0.0032 was well below the 0.05 threshold, signifying the statistical significance of the regression equation. Additionally, the coefficient of determination (R^2^) exceeds 0.8, indicating a robust fit to the data and affirming the model’s ability to interpret the PB design’s results effectively.

The electrochemical dissolution of GH4738 scrap was markedly affected by four principal factors: current density, NiCl_2_ concentration, electrolysis duration, and acid concentration. These factors contribute positively to both dissolution rate and energy consumption. Their combined effects boost process efficiency, which may result in higher dissolution rates or more efficient energy use. As such, these variables were recognized as the main determinants of the electrochemical dissolution process. The response values of these factors were analyzed to establish the direction and magnitude of the steepest ascent path.

### 3.3. Path of the Steepest Ascent Method

The steepest ascent test, informed by the Plackett–Burman (PB) experiment outcomes, targeted regions with low energy consumption (E) and high dissolution rates (η). Four experimental gradients were established based on the factors’ directional changes. Table 6 details the experimental design and outcomes, highlighting Group 3′s highest comprehensive indicator (Z) value. Consequently, a central composite design for the Response Surface Methodology (RSM) was initiated with a current density of 650 mA/cm^2^, acidity of 1.5 mol/L, NiCl_2_ concentration of 50 g/L, and electrolysis time of 0.75 h.

### 3.4. Analysis and Results of Central Composite Design

Table 7 displays the input values for the four pivotal parameters: NiCl_2_ concentration, acidity concentration, current density, and electrolysis time, and their corresponding output responses, which are energy consumption and dissolution rate, for a subset of 11 out of the total 29 experimental data sets. Analysis of Variance (ANOVA) evaluated the impact of these parameters and their interactions on the responses. The *F*-value, derived from the ratio of Mean Square Between to Mean Square Error, affirms the assessment’s validity [14]. The *p*-value gauges the statistical evidence against the null hypothesis, with lower values indicating stronger evidence for rejection. It is a pivotal metric in hypothesis testing, aiding in the validation or refutation of the null hypothesis and in assessing the significance of model coefficients [15]. A *p*-value below 0.05 is generally regarded as statistically significant at the 95% confidence level. Degrees of freedom (*d_f_*) for subjects are calculated by subtracting the number of treatments from the total number of subjects, while error d*_f_* is found by subtracting the number of groups from the total number of subjects [16]. For dissolution rate (η), model terms A, D, AD, and C^2^ are significant; for energy consumption (E), model terms A, B, AB, BC, BD, A^2^, and D^2^ are significant. Values exceeding 0.1000 in Table 8 and Table 9 indicate that the corresponding model terms are not statistically significant. The final regression models are succinctly summarized below:

(7) $$\eta =55.60+14.79A+17.53D+5.68AD-2.38C^{2}$$

(8) $$E=3.39+0.6136A-0.1043B-0.2792AB-0.1380BC-0.2490BD-0.1641A^{2}-0.1118D^{2}$$

### 3.5. Influences of Laboratory Parameters on Responses

#### 3.5.1. Influence of NiCl_2_ Concentration

**Figure 1:**
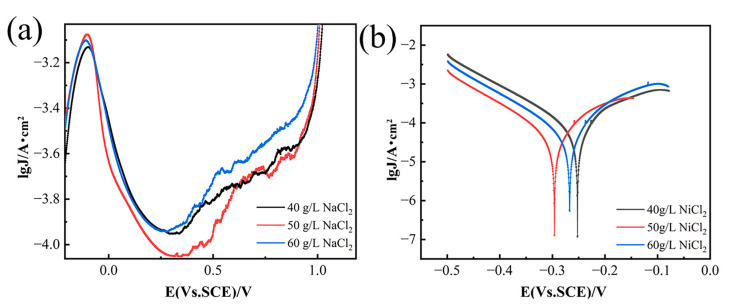
Anodic polarization curves of GH4738 scrap under different NiCl_2_. (**a**) Potentiodynamic polarization curves and (**b**) Tafel curves.

Figure 1a shows that increasing NiCl_2_ concentration shifts the anodic polarization curve towards higher current density values. This trend is due to a significant activation effect, which leads to a gradual rise in the passivation current density for nickel dissolution. The rapid increase in the anodic polarization curve is closely related to the continuous hydrogen evolution process [17,18]. Anodic dissolution in nickel-based alloys involves the formation, breakdown, and dissolution of the passivation film on the substrate’s surface [19]. Contrary to common assumptions, film breakdown sites do not align with high chloride ion concentrations but occur at adjacent areas where chloride ions have a lesser influence. Research indicates that passive films are mostly amorphous, with a small number of embedded nanocrystals (NCs), and the interfaces between NCs and the amorphous region resemble grain boundaries, facilitating chloride ion transport [20]. However, high NiCl_2_ concentrations increase the electrolyte’s internal stress, reducing the passivation current density. Thus, at a NiCl_2_ concentration of 50 g/L, the passivation current density is at its peak. As shown in Figure 1b, the breakdown potential (E_b_) shifts negatively with increasing NiCl_2_ concentration, and the anode slope decreases due to reduced reaction resistance, accelerating the corrosion rate. These observations underscore the effect of NiCl_2_ concentration on the passivation film’s breakdown.

**Figure 2:**
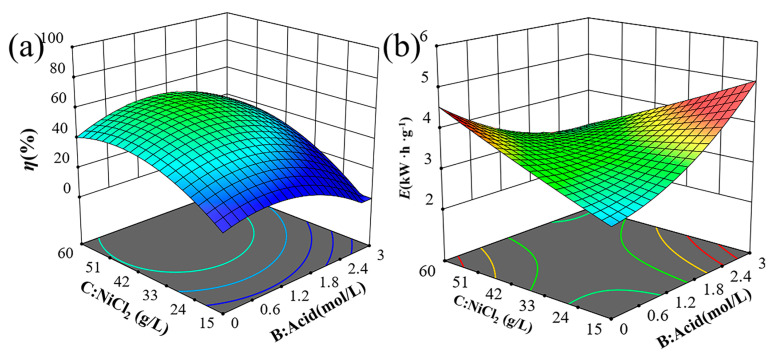
Trend graph of test indicators 3D surface between NiCl_2_ and H_2_SO_4_ concentration on (**a**) solubility and (**b**) energy consumption.

The 3D surface analysis is crucial for evaluating the parameters that influence the response. It helps to understand the interactions between NiCl_2_ concentration, current density, H_2_SO_4_ concentration, and electrolysis time on the response surface. Figure 2 illustrates the 3D surfaces of η and E for various combinations of NiCl_2_ and H_2_SO_4_ concentrations at a fixed current density and electrolysis time. Figure 2a indicates that η initially increases and then decreases with rising NiCl_2_ concentrations. High NiCl_2_ concentrations in the electrolyte increase ionic concentrations, enhancing ion interactions and increasing internal stress. This increased stress can impede ion migration and reactions, slowing the electrolytic process and reducing solubility. Figure 2b shows that E slightly decreases as NiCl_2_ concentration increases from 40 g/L to 50 g/L, but then rises rapidly as the concentration goes from 50 g/L to 60 g/L.

**Figure 3:**
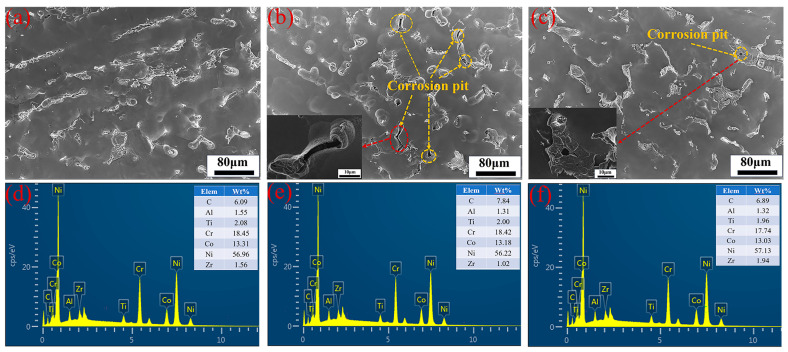
Surface appearance and EDX of GH4738 scrap under different NiCl_2_ concentration: (**a**,**d**) 40 g/L, (**b**,**e**) 50 g/L, and (**c**,**f**) 60 g/L.

Figure 3 displays the SEM images of the corroded surfaces after the removal of corrosion products for samples tested at an H_2_SO_4_ concentration of 1.5 mol/L, a current density of 650 mA/cm^2^, and an electrolysis time of 500 s, with varying NiCl_2_ concentrations: (a) 40 g/L, (b) 50 g/L, and (c) 60 g/L. The images reveal that corrosion becomes more severe with increasing NiCl_2_ concentration under identical test conditions. Figure 3a shows no visible corrosion pits, while Figure 3b clearly displays them. This observation aligns with the anodic polarization curves in Figure 1b, suggesting the presence of a protective film. The protective effect of the corrosion film is diminished by the presence of Cl^−^ ions, which convert Ni(OH)_2_ into soluble NiCl_2_, reducing the film’s protective properties. The anodic polarization curves also indicate that as NiCl_2_ concentration increases, the breakdown potential decreases and the corrosion current density increases, providing further evidence of the reduced protective film properties. Figure 3c shows a reduction in corrosion pits, consistent with the findings in Figure 1b, where high NiCl_2_ concentrations increase ionic concentration, enhance ion interactions, and raise internal stress. Figure 3d–f depicts the mass percentage variations in metals across varying nickel chloride concentrations. At 40 g/L NiCl_2_, Al mass percentage is 1.55%, decreasing to 1.31% at 50 g/L, suggesting that higher concentrations may marginally increase aluminum’s dissolution rate, thereby reducing its mass percentage in the matrix. Ti mass percentage is stable between 2.00% and 2.08%, indicating its stability during electrochemical dissolution and insensitivity to NiCl_2_ concentration changes. Cr mass percentage drops from 18.45% at 40 g/L to 18.42% and 17.74% at 50 g/L and 60 g/L, respectively, which may indicate a heightened dissolution rate at elevated NiCl_2_ concentrations. Co mass percentage decreases slightly from 13.31% to 13.18% and 13.03%, implying a minor rise in its dissolution rate with increasing NiCl_2_ concentration. Ni, being the principal metal in GH4738 scrap, shows a mass percentage that first rises and then falls with increasing NiCl_2_ concentration, reflecting a variable reaction rate. Zr mass percentage exhibits significant fluctuations, from 1.56% down to 1.02% and up to 1.94%, indicating a dissolution rate that initially increases and subsequently decreases. While the dissolution rates of Al, Co, and Cr incrementally rise with NiCl_2_ concentration, those of Ni and Zr show an initial increase followed by a decrease. Given the experimental focus on the nickel dissolution rate, a NiCl_2_ concentration of 50 g/L is identified as the optimal condition.

#### 3.5.2. Influence of H_2_SO_4_ Concentration

**Figure 4:**
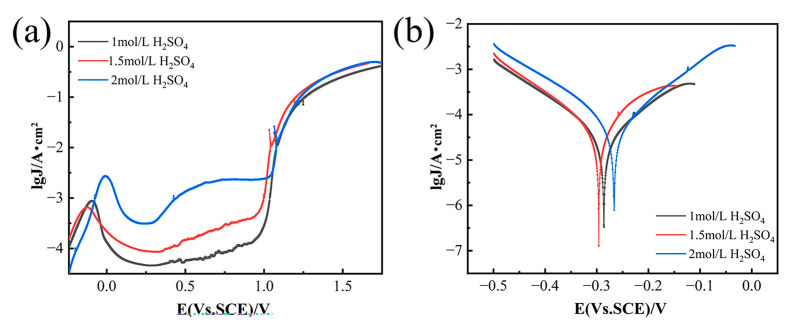
Anodic polarization curves of GH4738 scrap under different H_2_SO_4_ concentration. (**a**) Potentiodynamic polarization curves and (**b**) Tafel curves.

**Figure 5:**
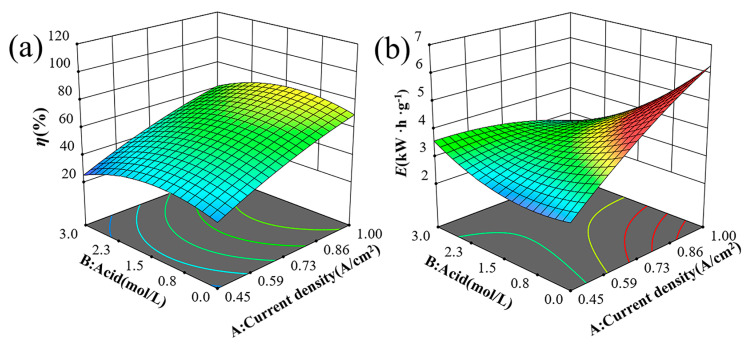
Trend graph of test indicators 3D surface between current density and H_2_SO_4_ concentration on (**a**) solubility and (**b**) energy consumption.

As depicted in Figure 4a,b, the pitting corrosion resistance (Φ), which is defined as the difference between the protective potential (E_tp_) and the breakdown potential (E_b_) [21], exhibits a decrease followed by an increase with the rise in NiCl_2_ concentration. The nickel anode demonstrates the lowest resistance to pitting corrosion in a 1.5 mol/L H_2_SO_4_ solution. During anodic polarization, current density decreases initially and then rises with increasing H_2_SO_4_ concentration, reaching a peak at 1.5 mol/L H_2_SO_4_. Figure 5 shows the effects of H_2_SO_4_ concentration and current density on solubility and energy consumption, assuming constant NiCl_2_ concentration and electrolysis time. Figure 5a indicates that solubility (η) first increases and then decreases with increasing H_2_SO_4_ concentration. This trend is attributed to the higher hydrogen ion concentration in more acidic solutions, which promotes hydrogen gas formation. This process destabilizes the passivation film and enhances matrix dissolution. However, beyond a certain H_2_SO_4_ concentration threshold, solute molecules may become locally supersaturated, leading to frequent ion collisions that hinder movement and limit diffusion rates [22,23]. Figure 5b demonstrates that energy consumption (E) first decreases and then increases as H_2_SO_4_ concentration rises. The initial decrease in energy consumption is due to the higher frequency of ion collisions at increased H_2_SO_4_ concentrations, which speeds up the reaction rate and reduces the time required to achieve a similar level of reaction. Consequently, this leads to lower energy consumption. However, at very high H_2_SO_4_ concentrations, the reaction generates significant heat, necessitating additional energy for heat dissipation and maintenance of reaction temperature stability, thereby increasing energy consumption.

#### 3.5.3. Influence of Current Density and Electrolysis Time

**Figure 6:**
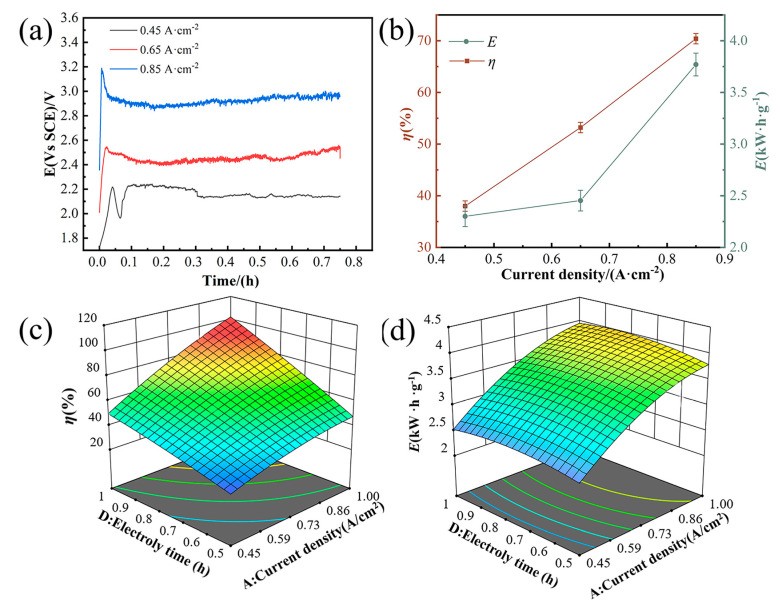
Current density and electrolysis time impact on dissolution and energy efficiency. (**a**) Current–density curves at a set electrolysis duration. (**b**) Variation in the dissolution rate and energy consumption across current densities. (**c**) 3D trend analysis: solubility as a function of current density and electrolysis time. (**d**) 3D trend analysis: energy consumption as a function of current density and electrolysis time.

Current density and electrolysis duration are pivotal parameters influencing dissolution rates and energy expenditure in electrochemical processes. Figure 6a depicts constant current curves at a set electrolysis duration, showing an increase in potential with rising current density. This trend is due to the intensified electrochemical reactions at the electrode surface, which also boosts the dissolution rate. Figure 6b further indicates that increasing current density correlates with higher dissolution rates and energy consumption. This pattern suggests that electrochemical reactions shift from being kinetically controlled at the electrode surface to being limited by mass transport at elevated current densities, consequently raising energy demands [24,25]. The interplay between current density and electrolysis time on dissolution rates and energy consumption is highlighted in Figure 6c,d. Prolonging the electrolysis time allows for dissolution rates and energy consumption to approach levels typically seen at higher current densities, even when operating at lower current densities. This underscores the substantial impact of electrolysis time on the dissolution process, emphasizing the need to consider both current density and electrolysis time when optimizing electrochemical processes.

### 3.6. Optimization and Confirmation

In this part, we utilized the Design Expert 13.0.5.0 for numerical optimization, selecting parameters and responses from the menu to meet our objectives. These objectives included setting a range, targeting a specific value, minimizing or maximizing responses, and specifying exact parameter values. Parameters were set within their experimental ranges to optimize the dissolution rate and minimize energy consumption, with the most favorable outcomes being higher dissolution rates and lower energy usage. The regression and experimental data were compared to calculate the percentage error, as detailed in Table 10. To validate the preferred dissolution process, we performed three replicate tests, and the results, with errors under 10%, confirmed the process’s stability and viability.

## 4. Conclusions

This study investigated the influence of complex laboratory factors on the electrochemical dissolution response of GH4738 scrap using statistical methods. The Plackett–Burman Design (PBD) effectively identified four significant factors influencing both dissolution rate and energy consumption: current density, NiCl_2_ concentration, electrolysis time, and H_2_SO_4_ concentration. Subsequently, the steepest ascent path method was employed to locate an optimal region characterized by high dissolution rates and low energy consumption, leading to a Response Surface Methodology (RSM) under specific conditions. The research revealed that elevated concentrations of NiCl_2_ and H_2_SO_4_ can impact the passivation film, thereby affecting the dissolution rate. Two quadratic models, developed using Response Surface Methodology (RSM), accurately predicted the parameters with high R^2^ values, indicating a strong fit between the model and the experimental data. Optimal conditions for achieving a high dissolution rate and minimal energy consumption were identified as a current density of 850 mA/cm^2^, an acidity level of 2.0 mol/L, a NiCl_2_ concentration of 60 g/L, and an electrolysis time of 1 h.

## Figures and Tables

**Table 1:** Composition of GH4738 scraps.

Element	Mo	Cu	Al	Ti	Fe	Co	Zr	Ni	Cr
Wt.%	4.25	0.1	1.4	3	2	13.5	0.05	58	19.5

**Table 2:** Factors and levels of Plackett–Burman design.

Factor	Level	
	−1	1
A Current density [A/cm^2^]	0.25	0.50
B H_2_SO_4_ concentration [mol/L]	0.5	1.0
C NiSO_4_ [g/L]	38.2	76.4
D NiCl_2_ [g/L]	6.7	10.0
E Na_2_S_2_O_3_ [g/L]	0.0158	0.0237
F Electrolysis time [h]	0.25	0.50
G Soaking time [s]	300	600
H H_3_BO_4_ [g/L]	5.50	10.91

**Table 3:** Factor and levels of Central Composite Design.

Factor	Level		
	−1	0	1
A Current density [A/cm^2^]	0.45	0.65	0.85
B H_2_SO_4_ concentration [mol/L]	1.0	1.5	2.0
C NiCl_2_ [g/L]	40	50	60
D Electrolysis time [h]	0.5	0.75	1.0

**Table 4:** Results of the Plackett–Burman Design.

Run	A Current Density [A/cm^2^]	B H_2_SO_4_ Concentration [mol/L]	C NiSO_4_ [g/L]	D NiCl_2_ [g/L]	E Na_2_S_2_O_3_ [g/L]	F Electrolysis Time [h]	G Soaking Time [s]	H H_3_BO_4_ [g/L]	*η* [%]	*E* [kW·h·g^−1^]
1	−1	1	−1	1	1	−1	1	1	6.4	1.950
2	1	1	−1	−1	−1	1	−1	1	20.9	3.116
3	1	1	1	−1	−1	−1	1	−1	14.6	2.410
4	1	1	−1	1	1	1	−1	−1	29.7	3.110
5	−1	−1	−1	−1	−1	−1	−1	−1	8.4	2.039
6	1	−1	1	1	−1	1	1	1	25.1	3.782
7	−1	−1	1	−1	1	1	−1	1	15.4	2.171
8	1	−1	1	1	1	−1	−1	−1	10.9	3.517
9	−1	1	1	−1	1	1	1	−1	12.2	2.040
10	1	−1	−1	−1	1	−1	1	1	14.6	3.085
11	−1	1	1	1	−1	−1	−1	1	9.0	1.766
12	−1	−1	−1	1	−1	1	1	−1	17.2	1.617

**Table 5:** Variance analysis of Plackett–Burman Design results.

Factor	SS	MS	F	Significance
model	473.47	94.69	9.27	
A Current density [A/cm^2^]	185.65	185.65	18.17	2
B sulfuric H_2_SO_4_ concentration [mol/L]	0.12	0.12	0.012	3
C NiSO_4_ [g/L]	8.33	8.33	0.820	5
D NiCl_2_ [g/L]	124.00	124.0	12.100	4
E Na_2_S_2_O_3_ [g/L]	3.00	3.00	0.160	8
F Electrolysis time [h]	266.96	266.96	26.12	1
G Soaking time [s]	1.47	1.47	0.078	7

**Table 6:** Result of the steepest accent experiments.

Run	A Current Density [A/cm^2^]	B H_2_SO_4_ Concentration [mol/L]	C NiCl_2_ [g/L]	D Electro-Lysis Time [h]	η [%]	E [kW·h·g^−1^]	Z
1	0.25	0.5	6.7	0.25	8.3	1.845	0.470
2	0.45	1.0	40	0.50	28.8	2.155	0.478
3	0.65	1.5	50	0.75	60.5	2.456	0.555
4	0.85	2.0	60	1.00	97.0	3.460	0.530

**Table 7:** Experimental parameters in actual units and experimental responses (11 out of 29 Data Sets).

Run	Parameters				Responses	
	A Current Density [A/cm^2^]	B H_2_SO_4_ Concentration [mol/L]	C NiCl_2_ [g/L]	D Electrolysis Time [h]	η [%]	E [kW·h·g^−1^]
1	0	0	−1	−1	35.1	3.245
2	0	−1	0	−1	36.3	3.132
3	1	0	−1	0	60.8	3.913
4	1	0	0	−1	46.3	3.635
5	0	0	1	−1	35.4	3.168
6	−1	0	0	1	47.9	2.525
7	1	0	0	1	92.0	3.760
8	−1	0	0	−1	24.9	2.486
9	0	1	1	0	52.7	3.122
10	0	0	0	0	51.0	3.164
11	−1	0	−1	0	38.3	2.547

**Table 8:** Analysis of Variance of the quadratic polynomial model for η (dissolution efficiency).

Source	d*_f_*	F-Value	*p*-Value	
Model	14	71.62	<0.0001	significant
A-current density	1	402.85	<0.0001	
B-H_2_SO_4_ concentration	1	2.95	0.1077	
C- NiCl_2_	1	1.11	0.3108	
D- Electrolysis time	1	566.03	<0.0001	
AB	1	0.0035	0.9540	
AC	1	1.25	0.2831	
AD	1	19.77	0.0006	
BC	1	0.3686	0.5535	
BD	1	0.2209	0.6456	
CD	1	0.2796	0.6052	
A^2^	1	3.05	0.1027	
B^2^	1	1.35	0.2655	
C^2^	1	5.61	0.0327	
D^2^	1	1.40	0.2559	

**Table 9:** Analysis of Variance of the quadratic polynomial model for E (energy consumption).

Source	d*_f_*	F-Value	*p*-Value	
Model	14	38.44	<0.0001	significant
A-current density	1	433.54	<0.0001	
B-H_2_SO_4_ concentration	1	12.51	0.0033	
C- NiCl_2_	1	0.0063	0.9380	
D- Electrolysis time	1	1.85	0.1958	
AB	1	29.93	<0.0001	
AC	1	1.29	0.2749	
AD	1	0.18	0.6814	
BC	1	7.31	0.0171	
BD	1	23.80	0.0002	
CD	1	0.3228	0.5789	
A^2^	1	16.77	0.0011	
B^2^	1	1.33	0.2689	
C^2^	1	0.0452	0.8346	
D^2^	1	7.78	0.0145	

**Table 10:** Parameters and Results of the Confirmation Experiment.

NO.	Parameters				Responses					
	A	B	C	D	η (%)			E (kW·h·g^−1^)		
					EXP	Regress-Ion	Error%	EXP	Regress-Ion	Error%
1	0.85	2	60	1	86.9	88.7	2.07	3.202	3.035	5.22
2	0.85	2	60	1	87.0	88.7	1.96	3.278	3.035	7.41
3	0.85	2	60	1	85.9	88.7	3.22	3.358	3.035	9.62

## Data Availability

The original contributions presented in this study are included in the article. Further inquiries can be directed to the corresponding author.
